# Expression profiling of a high-fertility mouse line by microarray analysis and qPCR

**DOI:** 10.1186/1471-2164-9-307

**Published:** 2008-06-27

**Authors:** Jens Vanselow, Gerd Nürnberg, Dirk Koczan, Martina Langhammer, Hans-Jürgen Thiesen, Norbert Reinsch

**Affiliations:** 1 Forschungsinstitut für die Biologie landwirtschaftlicher Nutztiere (FBN), Wilhelm-Stahl-Allee 2, 18196 Dummerstorf, Germany; 2 Institut für Immunologie, Universität Rostock, Schillingallee 70, 18055 Rostock, Germany

## Abstract

**Background:**

In a recent study it was demonstrated that a largely increased ovulation number is responsible for high prolificacy in two mouse lines selected for fertility performance. The objective of the present study was to identify genes that are involved in increasing the ovulation number in one of these lines, FL1. For differential expression profiling, ovaries of FL1 and of a non-selected control line, DUKsi, both lines derived from the same genetic pool, were analyzed with microarray analysis and quantitative polymerase chain reaction (qPCR). Ovaries from 30 animals of each line were collected at the metestrous stage, combined to 6 pools each, and processed for microarray analysis.

**Results:**

The actual number of ova shed in FL1 exceeded that of the DUKsi control line more than twofold (26.6 vs. 12.9). 148 differentially expressed ovarian transcripts could be identified, 74 of them up- and 74 down-regulated. Of these, 47 significantly mapped to specific Gene Ontology (GO) terms representing different biological processes as steroid metabolism, folliculogenesis, immune response, intracellular signal transduction (particularly of the G protein signaling cascade), regulation of transcription and translation, cell cycle and others. qPCR was used to re-evaluate selected transcripts and to estimate inter-individual variation of expression levels. These data significantly correlated with microarray data in 12 out of 15 selected transcripts but revealed partly large variations of expression levels between individuals.

**Conclusion:**

(1) The abundance of numerous ovarian transcripts was significantly different in FL1 compared to the non-selected control line DUKsi thus suggesting that at least some of the respective genes and corresponding biological processes are involved in improving reproductive traits, particularly by increasing the number of ovulation. (2) Selective qPCR re-evaluation largely confirmed the microarray data and in addition demonstrated that sample pooling can be beneficial to find out group-specific expression profiles despite of large inter-individual variation. (3) The present data will substantially help ongoing genetic association studies to identify candidate genes and causative mutations responsible for increased fertility performance in mice.

## Background

In polytocous species as mouse and pig the number of ova shed is an important parameter for reproductive performance. Folliculogenesis, ovulation, and luteinization are regulated through endocrine and paracrine feedback mechanisms along the hypothalamo-pituitary-gonadal axis. Important endocrine factors are the hypothalamic gonadotropin releasing hormone, follicle stimulating hormone (FSH) and luteinizing hormone from the pituitary gland, and steroid and peptide hormones generated within the ovary as estrogen, progesterone, inhibin, activin, and follistatin. Within the ovary also paracrine, locally acting hormones and growth factors of the IGF and TGF-β families play an important role during folliculogenesis and ovulation [1]. In time production, storage, release, but also cease of production and release of these regulatory factors require a well orchestrated activation and de-activation of genes that are involved in this regulatory network. In general, reproductive traits show a relatively low heritability and are strongly affected by environmental factors. Nonetheless it could be demonstrated in pig and mouse that selection on litter size can effectively improve the reproductive performance and particularly affects the ovulation number but also other reproductive traits [2-6]. Because genetic factors are clearly involved in reproductive performance one must hypothesize that the expression levels of many genes that are involved in the regulatory network of reproduction are different in animals with different fertility performance. Therefore differential expression profiling of reproductive organs, mainly the ovary, is an appropriate approach to elucidate the genetic and physiological background of enhanced female reproductive performance. In our recent study it could be clearly demonstrated that a largely increased ovulation number is mainly responsible for high prolificacy in selected high fertility mouse lines [7]. Mining differentially expressed ovarian genes will help to elucidate the genetic and physiological consequences of selection for reproductive performance but might also help to better understand regulatory pathways that are important for folliculogenesis and successful ovulation.

During the present study a high fertility mouse line, FL1, which was generated by index trait selection over more than 130 generations [7] was comparatively analyzed with the non-selected control line, DUKsi. To screen for differentially expressed ovarian genes, staged ovaries of both lines were screened for differentially abundant transcripts. Both lines have been derived from the same genetic pool. This should minimize the number of genetic differences that are only due to non-trait related line-specific differences. The ovarian expression profile of both lines was analyzed by microarray analysis. To include a large number of individuals on one hand and to avoid excessive microarray costs on the other hand, 30 animals of each line were combined to six pools. Transcript abundance of selected genes were re-evaluated with quantitive real-time PCR (qPCR) in pooled as well as individual samples to estimate inter-individual variation.

## Results

### Fertility traits

After 130 generations of selection, fertility performance was largely and significantly increased in FL1. The mean number of corpora lutea at the first day of pregnancy was 18.2 ± 6.8 (mean ± std, n = 10) compared to 12.2 ± 5.5 (n = 10) in the control line (p = 0.0437). The actual number of ova flushed from the oviducts however was even higher, 26.6 ± 15.4 in FL1 and 12.9 ± 3.2 in the control (p = 0.0131), thus exceeding the corresponding number of CL, particularly in FL1. This suggests that individual follicles can release more than one oocyte [7]. In fact, the existence of multioocyte follicles has been found in mice [8] but also in other species as goat [9]. The mean litter size and litter weight at birth increased during the selection period to 17.3 ± 7.7 (n = 60) and 27.8 ± 4.6 g, respectively in FL1 compared to 9.81 ± 2.03 (n = 75) and 15.1 ± 6.2 g in the control line (p < 0.0001 for litter size and weight). The body weight of individual newborn pups however was not significantly reduced in FL1 despite of the strongly increased number of pups per litter.

### Microarray analysis

For differential expression profiling, RNA preparations of 30 animals of each line were combined to 6 sample pools with 5 individuals per pool. After labeling, the cRNA of pooled samples was hybridized to MOE 430A microarrays, scanned and statistically evaluated. The following thresholds were used to define differentially expressed probe sets:

1. Transcript abundance was considered to be different in FL1 when it was at least 1.5 fold higher or lower compared to that in DUKsi; -fold change (FC) ≥ 1.5 or ≤ -1.5

2. False Discovery Rate: q -values < 0.01

3. > 60% of the pairwise comparisons (each of the six microarrays of FL1 was compared with each of the six microarrays of line DUKsi, n= 36 pairwise comparisons) should lead to one of both statements: increased or decreased

**Figure 1:**
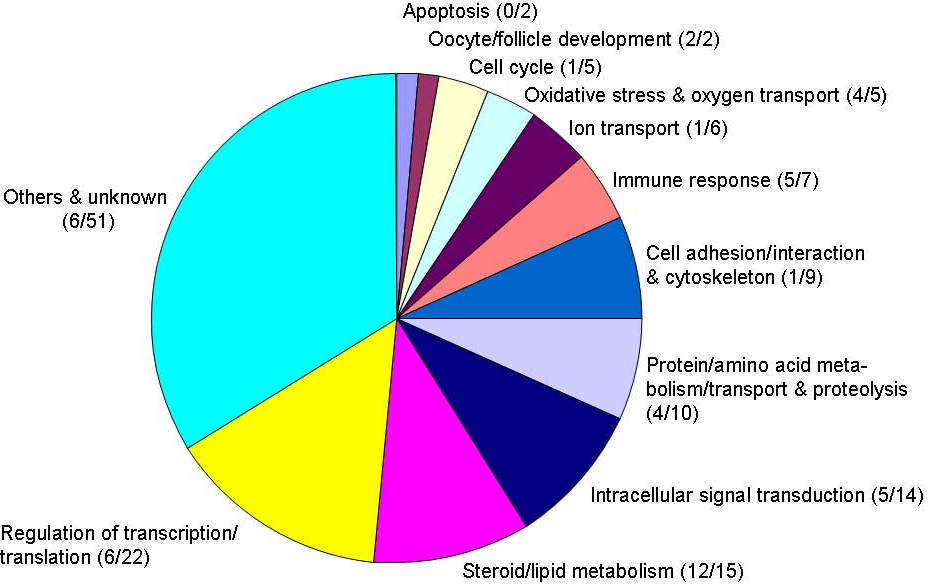
**Superordinate categories of biological processes identified by gene ontology (GO) mapping.** Differentially expressed transcripts mapped to numerous biological processes of the hierarchical GO system. These were combined to the shown eleven superordinate categories. The first number in brackets (preceding the slash) represents the number of transcripts, which significantly map to specific biological processes of the hierarchical GO system. The second number (following the slash) represents all transcripts mapping to the specified biological process without statistical significance.

All together, 1604 of the probe sets passed the first threshold (FC ≥ 1.5 or ≤ -1.5). 327 of these showed q-values < 0.01. The third threshold finally reduced the number of differentially expressed probe sets to 191. These represented only 148 distinct transcripts because some transcripts were represented by more than one probe set on the MOE 430A microarray. Of the differentially expressed transcripts 50% were up- (FC ≥ 1.5) and 50% were down-regulated (FC ≤ -1.5) in FL1 compared to the control. The FC values varied between -9.1 to -1.5 and 1.5 to 9. 125 (84%) of the identified transcripts could be assigned to known genes, but only 47 (32%) of them mapped significantly (p < 0.05) to 231 distinct GO terms representing biological processes. For the sake of clarity, related GO terms were combined to eleven superordinate categories (Fig. 1). All 148 differentially expressed transcripts, their respective gene symbols and gene titles, FC values and respective superordinate categories of biological processes are listed [see Additional file 1]. Most significantly, genes mapped to biological processes of the steroid and/or lipid metabolism. But also other processes as immune response, regulation of transcription and translation, and intracellular signal transduction with most genes mapping to G-protein related GO terms, were significantly affected.

### Re-evaluation of microarray data with real-time PCR

Selected, differentially expressed transcripts were re-analyzed with qPCR. Besides re-evaluation of the pooling approach, transcript concentrations were also determined in ten individual samples of each line to estimate inter-individual variation. Altogether, 15 probe sets were selected for qPCR re-evaluation according to the following criteria: (1) probe sets should preferably represent transcripts of well defined genes; (2) selected transcripts should cover a wide range of transcript abundance; (3) about half of them should be up and half of them down regulated in FL1; (4) probe sets should map to different GO terms.

**Figure 2:**
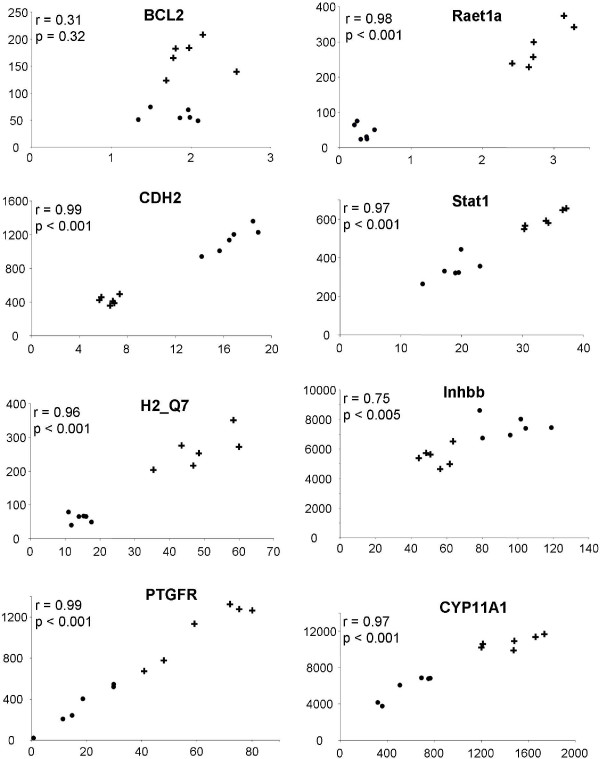
**Scatter blots showing the correlation of selected microarray and qPCR data from sample pools.** Ordinates indicate arbitrary intensity levels of expression microarray analyses; abscissae indicated transcript abundance as determined by qPCR analysis (copies × 10^5^/μg RNA). r, coefficient of correlation; p, p value calculated by the Pearson Product Moment correlation test.

**Figure 3:**
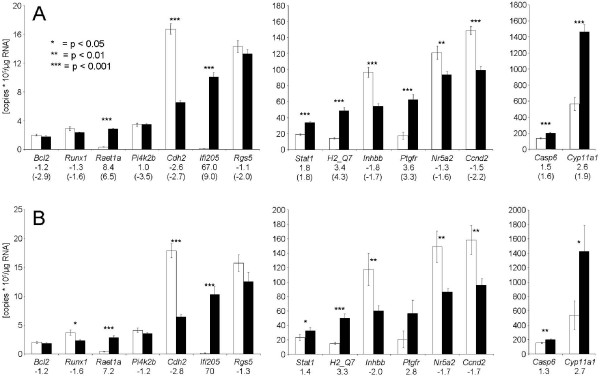
**Abundance of ovarian transcripts in FL1 and in the control line DUKsi.** Transcript abundance was determined by qPCR from (n = 6) pooled (A) and (n = 10) individual samples (B). Mean abundance and standard deviations are shown. Numbers indicate -fold change (FC) values calculated from qPCR data, numbers in brackets those calculated from microarray data [see Additional file 1].

For re-evaluation of the pooling approach, the samples were freshly assembled to 6 pools of each line, as described for microarray analysis. The qPCR data showed a strong and significant (p < 0.05) correlation with microarray data in twelve of the fifteen transcripts (Fig. 2). Three of the transcripts however, *Bcl2*, *Pi4k2b *and *Rgs5*, did not show any association between microarray and qPCR data. A comparison of the mean values that were calculated from all six pools of each line revealed that only eleven of the fifteen transcripts showed significantly different expression levels in both lines. Obviously, these differences between microrray and qPCR analysis were only found in transcripts with generally low expression levels. The relative transcript abundance (i.e. the transcript abundance determined in FL1 divided by that found in the control line) varied between FC values of -1.3 to 67 (Fig. 3A). Generally, these relative expression levels were similar to those found by microarray analysis. Only in case of Ifi205 the FC value calculated from qPCR data was much higher than that calculated from microarray data (FC 67 vs. FC 9, see Fig. 3A). This might be due to the limited dynamic range of the hybridization technique used for microarray analysis compared to the range of qPCR, which can exceed more than 5 orders of magnitude.

**Figure 4:**
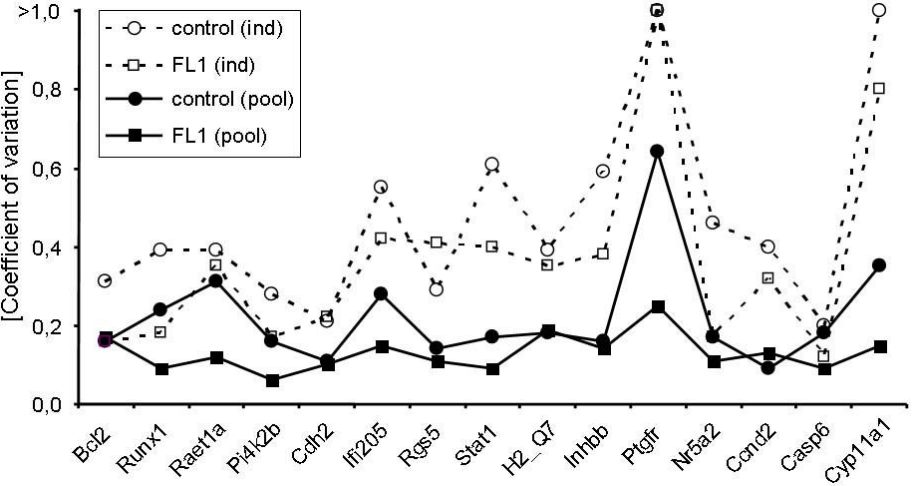
**Coefficients of variation of transcript abundance calculated from qPCR data.** Transcript abundance was determined in pooled (solid lines) and individual samples (broken lines) from FL1 (squares) and the control line DUKsi (circles). Transcripts are ordered from low (left) to high levels (right) of expression. ind, individual samples; pool, pooled samples.

qPCR analysis of individual samples showed similar line-specific differences as the qPCR pooling approach (Fig. 3B). However, line-specific inter-individual variations were considerably higher than line-specific inter-pool variations. Thus the coefficients of variation of qPCR data from individual samples were higher throughout all levels of expression than those from pooled samples (Fig. 4). This also resulted in partially higher p values (t-tests) and thus lower levels of significance when comparing mean values of both lines.

## Discussion

During the present study comparative whole genome expression profiling was used to identify ovarian genes, whose expression pattern was affected by long term selection towards increased fertility performance. Mapping of these genes to the hierarchical GO terms could elucidate biological processes that might be responsible for the largely improved fertility performance in FL1. It cannot be completely excluded however, that some of these line-specific differences are due to undirected allelic drift as a consequence of genetic separation of both lines over many generations. In any case, the data from the present expression profiling approach will also crucially support ongoing association studies to clearly identify candidate genes and causative polymorphisms, which are actually responsible for the largely improved fertility performance in FL1.

### Microarray expression profiling

Numerous of the differentially expressed genes, which had been identified by microarray expression profiling are known to be involved in female reproductive processes as folliculogenesis, ovulation, atresia, luteinization, or luteal regression and thus might play a role in increasing the ovulation number. Hereafter, a selection of these genes will be discussed:

B-cell CLL/lymphoma 2 (*Bcl2*) and caspase 6 (*Casp6*), can be assigned to the process of apoptosis. Members of the *Bcl2 *and *Casp *families are well known to play a fundamental role during follicular atresia [10,11], but also throughout earlier stages of folliculogenesis [12].

Inhibin β-B (*Inhbb*), which was found down regulated in FL1 ovaries compared to those of the control, significantly maps to GO terms related to oocytes and follicle development. It is an important negative regulator of FSH release and thus is essentially involved in the regulation of folliculogensis and estrous cycle [13].

Cyclin D2 (*Ccnd2*) is known to be essentially involved in granulosa cell differentiation. The gene is induced by FSH and regulates cell proliferation in gonads [14]. Consequently, the absence of a functional gene in -/- mutant females leads to sterility [14]. Down-regulation of *Ccnd2 *by dihydrotestosterone induces cell cycle arrest in granulosa cells [15].

Glutathione peroxidase 3 (*Gpx3*) and peroxiredoxin 3 (*Prdx3*) are involved in the antioxidant defense and may play a role in protecting cells of the cumulus-oocyte complex but also luteal cells from oxidative stress-induced cell death [16-18].

Gulonolactone (L-) oxidase (*Gulo*) has also been found being expressed in the ovary [19]. It is involved in L-ascorbic acid biosynthesis, which can influence ovarian aromatase activity [20].

Seven genes were found to map to GO terms related to the immune system, five of them with significant p-values. Differential expression of three of them could be confirmed with qPCR. Histocompatibility 2 K1 K region (*H2-K1*) and histocompatibility 2 Q region locus 7 (*H2-Q7*) are involved in the process of antigen presentation. A possible functional role of these genes for reproductive performance is not clear yet. Interferon activated gene 203 (*Ifi203*) and interferon activated gene 205 (*Ifi205*) are target genes of the JAK/STAT intracellular signaling pathway that can be activated by the cytokine gamma interferon, a major player of immune cell signaling. Alternatively, the JAK/STAT pathway can also be activated by insulin like growth factor 1 (IGF1) [21]. IGF1 however is an important growth factor during folliculogenesis. Therefore the IGF1 signaling pathway might be affected by selection for increased fertility. This view is supported by significant up-regulation of signal transducer and activator of transcription 1 (*Stat1*) another player of the IGF/JAK/STAT/Ifi pathway. As also shown by microarray analysis, but particularly by qPCR re-evaluation, *Ifi205 *is dramatically up-regulated in the high fertility line thus strongly suggesting a functional role of this gene for fertility performance.

Cadherin 2 (*Cdh2*), a member of the cadherin family might play a functional role similar to N-cadherin, which was found to be expressed in granulosa cells and oocytes mediating cell to cell contacts [14].

GTP cyclohydrolase 1 (*Gch1*), guanine nucleotide binding protein β 1 (*Gnb1*), guanine nucleotide binding protein β 4 (*Gnb4*), Guanylate cyclase 1 soluble α 3 (*Gucy1a3*), Guanylate cyclase 1 soluble β 3 (*Gucy1b3*), IQ motif containing GTPase activating protein (*Iqgap2*), prostaglandin F receptor (*Ptgfr*), and regulator of G protein signaling 5 (*Rgs5*) are involved in the G protein signaling pathways. This suggests that G protein signaling was actually affected by selection towards high fertility and might be important for increased reproductive performance in FL1.

A large group of genes maps to GO processes of the lipid and particularly steroid metabolism. Steroids are important regulators of folliculogenesis and luteal differentiation. Transcript levels of the niemann pick type C2 (*Npc2*) gene that is involved in sterol trafficking, and of the *Cyp11a1 *gene encoding the cholesterol side-chain cleavage enzyme, which catalyzes an initial step of steroid hormone synthesis, were found to be up-regulated in FL1. Most interestingly, also the expression of an important transcriptional regulator of steroid biosynthesis, the nuclear receptor subfamily 5 group A member 2 (*Nr5a2*) was found significantly changed in ovaries of FL1. *Nr5a2*, also known as liver receptor homologue 1 (*Lrh1*), shows high level expression in ovarian cells. It is regulated by FSH, luteinizing hormone (LH) and prolactin, and is known to regulate important steroidogenic genes as *Star*, *Cyp11a1*, *Cyp17*, *Hsd3b*, or *Cyp19 *[22-24]. *Nr5a2 *knockout is lethal in homozygotes and leads to reduced female fertility in heterozygote animals [25], most likely because of altered luteal function due to reduced progesterone production. This suggests that steroid hormone production was affected by selection towards increased fertility performance. However, there is no conclusive explanation for the contradictory observation that expression of *Nr5a2 *is up-, but that of *Cyp11a1 *is down-regulated in FL1, in spite of the fact that *Nr5a2 *is a positive regulator of steroidogenic genes.

It is well known that cAMP responsive element binding proteins are essentially involved in FSH signaling in ovarian granulosa cells [26]. Expression of the cAMP responsive element binding protein 3 (*Creb3*) was found largely up-regulated in ovaries of FL1 thus suggesting, that the FSH signaling cascade that is essential for follicular growth and differentiation might be affected, possibly enhanced, by selection towards increased fertility. Another interesting transcriptional regulator, which might influence reproductive performance, is the RAR-related orphan receptor alpha gene (*Rora*). Mutations of this gene result in the "staggered" phenotype in mice, which is characterized by cerebellar abnormalities. In addition, "staggered" mothers produce smaller litters and a reduced number of oocytes thus indicating pleiotropic effects of *Rora *on fertility performance [27]. Expression of the runt related transcription factor 1 (*Runx1*) in follicular cells has been well documented. This factor seems to play an important role in the preovulatory process by regulating LH induced progesterone production [28]. However, significantly reduced expression of *Runx1 *transcripts in ovaries of FL1 animals could not be unambiguously confirmed by qPCR.

A member of the retinol dehydrogenase family, *Rdh2*, was identified by microarray analysis in polycystic ovary syndrome theca cells [29], where it affects steroid synthesis. Over expression of *Rdh11*, that was found four-fold up-regulated in ovaries of FL1 thus suggests again, that steroid synthesis has been affected by selection towards improving fertility performance. Stanniocalcin 1 (*Stc1*) is known to be expressed in thecal interstitial cells. This paracrine hormone binds to receptors that are located in the granulosa cell layer, where it may act as a luteinization inhibitor [30]. *Stc1 *knockout experiments however revealed, that this gene is not essential for reproduction and development in mice [31], but it may modulate follicular differentiation and may therefore be important for reproductive performance.

### Re-evaluation by qPCR

By qPCR re-evaluation the majority of microarray data could be confirmed. However in three out of the 15 genes, qPCR data do not correlate with microarray data. Noticeably, two of these genes, *Bcl2 *and *Pi4k2b*, showed very low transcript abundance levels. Probably, these levels may fall below a technical threshold and therefore do not allow a reliable transcript quantification particularly by hybridization based methods as microarray analysis. Thus expression data at these particularly low levels should be interpreted with care. Microarray and qPCR data of other transcripts however showed significant correlations, particularly those with intermediate and high expression levels. Therefore this re-evaluation indicated that the microarray based differential expression profiling basically yielded reliable data.

In addition to re-evaluation, qPCR was also used to estimate inter-individual variability of transcript abundance. The pooling approach applied for qPCR was identical to that for microarray analysis. The line-specific mean transcript levels were similar in both experimental approaches. However, the inter-individual variability was much larger than the variability between different sample pools of the same line. Particularly *Cyp11a1*, *Ptgfr*, and *Inhbb*, but also *Nr5a2 *and *Ccnd2 *showed very large inter-individual variability. Remarkably, this inter-individual variability was especially distinct in the control line. Thus far however, we have no well-founded explanation for this observation. As a consequence of this very large inter-individual variability, the significance of comparative statistics (t-test) was considerably lower (e.g. *Stat1*, *Inhbb*, *Nr5a2*, *Ccnd2*, *Casp6*, *Cyp11a1*). In case of the prostaglandin F receptor (*Ptgfr*), despite of very different mean expression levels between both lines, this difference was only significant when sample pools instead of individual samples were compared. This suggests that large inter-individual variation of expression levels can obscure group-specific differences. This is in line with an earlier observation [32] stating that pooling is beneficial when many subjects, i.e. more than 2 to 3, are pooled.

## Conclusion

(1) The ovarian expression levels of numerous genes, which are involved in steroid/lipid metabolism, immune response, G-protein signaling and other biological processes were significantly different in the selected high fertility mouse line FL1 compared to a non-selected control line. This suggests that at least some of these genes and the corresponding biological processes play a functional role in improving reproductive traits, particularly by increasing the number of ovulation.

(2) Selective qPCR re-evaluation largely confirmed the microarray data and in addition demonstrated that sample pooling allows the inclusion of more individuals into expression profiling analysis with simultaneous reduction of costs and can be beneficial to find out group-specific expression profiles despite of large inter-individual variation.

(3) Combined with ongoing association studies in a FL1/DUKsi crossbreeding family the present data will substantially help to identify candidate genes and causative mutations that are eventually responsible for increased fertility performance in mice.

## Methods

### Selection Procedure

Animal experiments were approved by the respective ethical authorities from the Land Mecklenburg-Vorpommern, Germany, in compliance with the European legislation on the care and use of laboratory animals.

All mouse lines were originally derived from the same genetic pool by systematic crossbreeding of 4 inbred and 4 outbred lines [33,34]. From this outbred population the high fertility line FL1 was generated during a long term selection experiment [35]. The line was selected for an index trait, combining litter size (LS0) and litter weight (LW0) at birth in primiparous females (Index (I) = 1.6 × LS0 + LW0)) to generation 130. The line was maintained with 60 to 100 mating pairs per generation. Line DUKsi is an inbred line that was not selected for any specific trait but originated from the same genetic pool as FL1. DUKsi was inbred by full-sib mating for about 36 generations. Females were mated at an age of 63 days with a mating ratio of 1:1. The mice were kept in Makrolon-cages (Ehret, Emmendingen, Germany) of 30 × 12.5 × 12.5 cm and had free access to pellet concentrate and water. The light regime was LD 12:12, the room temperature was standardized to 22.5°C.

### Determination fertility traits

To determine component fertility traits, the number of corpora lutea and of ova shed was analyzed in 10 animals of each line at the first day of pregnancy. Nine week old virgin females of each line were mated to males of the same line. Upon detection of a vaginal plug females were removed from mating cages. The day of plug detection was assigned as the first day of pregnancy. Animals were killed by decapitation and uteri with ovaries were removed from both sides. Ova, which frequently are still surrounded by cumulus cells, were harvested from the upper part of the oviduct, the ampulla, and incubated in 3% hyaluronidase in M2 medium (Sigma-Aldrich, Taufkirchen, Germany) for 3 to 5 minutes at room temperature, in order to release all ova from surrounding cumulus cells. Additional ova, separate to those in the cumulus-oocyte-complex, were flushed from the oviduct with M2 medium. The number of CL was counted under a microscope after dissection of both ovaries from every animal.

### Microarray expression profiling

For microarray expression profiling, ovarian RNA samples from 30 virgin females (six to nine weeks old) of each line were combined to six pools with five individuals per pool. Animals were selected from 25 and 26 different litters of FL1 and of the control, respectively, in order to reduce individual maternal effects. Samples from individuals were combined to pools by considering the respective size of litters where the animals descended from in order to generate pools from animals with a similar mean parental fertility performance (Tab. 1). One-way analysis of variance (ANOVA) revealed no significant differences of means between pools from the same line. In FL1, the size of litters ranged from 10 to 21 and in the control from 6 to 12.

**Table 1:** Mean size and standard deviation (std) of litters, from which animals have been selected for pooling.

	control		FL1	
	mean	std	mean	std
pool 1	9.8	3.5	16.0	5.5
pool 2	9.8	0.8	15.2	3.3
pool 3	10.0	1.6	16.8	1.9
pool 4	10.0	1.6	16.8	1.9
pool 5	10.4	1.1	16.8	1.6
pool 6	10.4	0.5	17.8	1.6
all	10.1	1.7	16.6	2.8

Ovaries were collected from females at the metestrous stage. Stages of the estrous cycle were determined between seven thirty and eight a.m. from serial vaginal smears with microscopic evaluation. Animals were killed two to three hours later, ovaries were dissected and freed from attached tissues, and preserved in RNAlater^® ^(Qiagen, Hilden, Germany). Right and left side ovaries were processed together for total RNA preparation using the RNeasey Mini Kit with simultaneous removal of genomic DNA with RNAse free DNAse (both from Qiagen). RNA was quantified in a GeneQuant II photometer (Pharmacia Biotech, Freiburg, Germany). Two μg RNA from each sample were then combined to pools with five samples and 10 μg RNA per pool.

First-strand synthesis was carried out by a T7-(dT)24 primer and SuperScript II Reverse Transcriptase (Gibco BRL Life Technologies) using ten μg pooled total RNA. Second-strand synthesis was done according to the SuperScript Choice System (Invitrogen) by E. coli DNA-Polymerase I, E. coli Ligase and RNaseH. Fragment end-polishing was performed using T4-Polymerase. An in vitro transcription reaction was used to incorporate Biotin-11-CTP and Biotin-16-UTP into the cRNA probe (BioArray HighYield RNA Transcript Labeling Kit, Enzo). The fragmented cRNA was hybridized overnight (45°C) to the Affymetrix MOE 430A mouse expression arrays (~20,000 probe sets = mouse gene specificities). Each sample pool was hybridized to two arrays to average technical variation. Arrays were then washed using the GeneChip Fluidics Station (Affymetrix) according to the manufacturer's protocol and stained by R-Phycoerythrin Streptavidin (Molecular Probes). This was followed by an antibody amplification procedure using a biotinylated anti-streptavidin antibody (Vector laboratories) and goat IgG (Sigma). The scanning was carried out with 3 μm resolution, 488 nm excitation and 570 nm emission wavelengths employing the GeneArray Scanner 2500 (Hewlett Packard). For calculation of signal intensity of each gene we used the Affymetrix Microarray Suite 5.0 Software (MAS5). All arrays were normalized by scaling to reach a common target intensity. Mean intensities of all probe sets were calculated from both technical replicates. Microarray data have been submitted to the Gene Expression Omnibus Database (GEO Series record: GSE11113).

As an initial statistical analysis a 'Comparison expression analysis' of MAS5 was done for all possible pairwise comparisons (n = 36) between six pool arrays of the DuKsi control line as baseline and 6 pool arrays of FL1 as experimental line. For each of the ~20,000 probe sets this resulted in one of the following statements: increased, decreased, not changed, or not detected. Additionally, the common t-test (MAS5) for each gene was performed to get a list of corresponding p-values of significance. Because of the high number of tested null hypotheses (22690) the number of false positive results was limited by using the concept of the false discovery rate (FDR). The FDR was calculated by means of the Q-value approach [36,37]. We used the software 'Q-value' written by Dabney/Storey – starting with the sorted list of p-values of the corresponding t-tests to get a list of estimated q-values. Distinct thresholds of Q-values, pairwise comparisons, and relative differences of mean expression levels between both lines (fold change, FC) were combined to define differentially expressed probe sets, respectively transcripts (see Results).

To map differentially expressed transcripts to specific biological processes of the hierarchical vocabularies of the Gene Ontology (GO) system, the GO Browser of the NETAFFX mining tool (Affymetrix) was used. Because some transcripts are represented by more than one probe set on the MOE 430A mouse expression arrays, redundant probe sets had to be removed from the list of differentially expressed probe sets before GO browsing. If the p-value that was calculated by the Chi square test for each GO term was below 0.05, GO mapping of the respective transcript was considered significant.

### qPCR

The abundance of selected transcripts, which had been previously identified by microarray expression profiling, was re-evaluated by qPCR in sample pools, and in addition, in ten individual samples of each line in order to estimate inter-individual variation of transcript levels. Pools for qPCR analysis were freshly prepared, however with the same samples and the same amount of RNA as described for microarray analysis. Primers used for reverse transcription and qPCR are shown in Tab. 2. Primers were designed to bind within or close to the target sequence of the corresponding microarray probe sets and if possible, were derived from different exons to avoid amplification of residual genomic DNA. For cDNA synthesis 0.1 μg total RNA were reversely transcribed in a 25 μl reaction volume using M-MLV reverse transcriptase, RNase H Minus, Point Mutant (Promega, Mannheim, Germany). The freshly synthesized cDNA samples were cleaned with the High Pure PCR Product Purification Kit (Roche, Mannheim, Germany) and eluted in 50 μl elution buffer. The identity of selected products generated with different primer pairs was controlled by sequencing.

**Table 2:** Primers used for cDNA synthesis and qPCR

**Gene**		**Sequenz**	**Accession nos**.	**bp**
*Bcl2*	rt	GGTCTGCACCTTTAATCCTAGTAC	BC027249	328
	rev	ACATACAGAGGCCTTGTCTCAGAC		
	for	GGAATGACATGTTGCTCACATTTAC		
*Casp6*	rt	TAGCCCTTCCACCACGTCCAAC	NM009811	231
	rev	GCGCTGAGAGACCTTTCTGTTC		
	for	ACAGACAAGCTGGACAACGTGAC		
*Ccnd2*	rt	CAGGCTTTGAGACAATCCACATC	NM009829	248
	rev	AATGAAGTCGTGAGGGGTGACTG		
	for	CTCTGGCCATGAATTACCTGGAC		
*Cdh2*	rt	CCCACCGCTACTGGAGGAGTTG	NM007664	204
	rev	GCCTCTCGTCTAGCCGTCTGA		
	for	GGATGAAACGGCGGGATAAAGAG		
*Cyp11a1*	rt	ATACAGAGATACCACCCTCAAATG	NM019779	252
	rev	TCACGGAGATTTTGAACTTCAAT		
	for	GATTCCAGCCAAGACTTTGGTAC		
*H2-Q7*	rt	CAATCAACCCTCAGCTCAAGATG	XM622842	293
	rev	TAGGCTCACAGGGAACATGAGAC		
	for	CTGAGCCTCTCACCCTGAGATG		
*Ifi205*	rt	GGTGACATTTCTATTTTGGCATCTC	NM172648	188
	rev	CCTTACAGTTGATGTTGTGCCATTG		
	for	CAGAAATGCAAATGCCAGTCCTAAG		
*Inhbb*	rt	ACCAGTGACCTGTCAGTTGTTG	XM148966	272
	rev	TGAGTCGCTCCTGGGCTACTTG		
	for	GCACTCTGAATTGCGCCTTCTGA		
*Nr5a2*	rt	CTTGGAGCAGTTCAGAGTATTGTG	NM030676	217
	rev	GTCTTCTGCCTGCTTGCTGATTG		
	for	CGATCAGCGGGAGTTTGTATGTC		
*Pi4k2b*	rt	CTGCACTCCACGATCACACATG	NM028744	203
	rev	AGCAGCTCTGTCAAATCCTTTGTC		
	for	GGCTAGCATTTCCCTTTAAGCATC		
*Ptgfr*	rt	AGTCCAGCTTCACTCGATGCTTG	NM008966	204
	rev	ACAGGTTCCTAAGGACAGCCTTC		
	for	TCATTCAGCTCCTGGCCATAATGT		
*Raet1a*	rt	AAATGTATTAGAGGAGGGAGATAAG	NM009016	218
	rev	CTCCAGTTCCACAGGATCCGATG		
	for	GGAAAAGCCAAGATCAACCTCAAG		
*Rgs5*	rt	ATGCAGCCCTTAGACTGCAGAAG	NM009063	242
	rev	GAGGCATCTGAGTGAGTGTGTAAC		
	for	GCTTCTAAACAGGATTCATTTCAATC		
*Runx1*	rt	TCGGAGATGGACGGCAGAGTAG	NM009821	246
	rev	GACAGAGGAAGAGGTGATGGATC		
	for	ATGAAGAACCAGGTAGCGAGATTC		
*Stat1*	rt	ATGTCGCCAGAGAGAAATTCGTG	NM009283	176
	rev	GGTGGACTTCAGACACAGAAATC		
	for	TGCCGAGAACATACCAGAGAATC		

bp, lengths of amplicons in base pairs; for, forward primer; rev, reverse primer; rt, primer used for cDNA synthesis

For qPCR, 0.5 and 0.25 μl of each purified cDNA sample were amplified with the LightCycler-FastStart DNA Master^PLUS ^SYBR Green I Kit (Roche, Mannheim, Germany) in 10 μl total reaction volume. Values from both reactions were averaged. Amplification and quantification of PCR products was performed in a LightCycler^® ^instrument (Roche) under the following cycling conditions: Pre-incubation at 95°C for 10 min, followed by 45 cycles denaturation at 95°C for 15 sec, annealing at 60°C for 10 sec, extension at 72°C for 10 sec and single point fluorescence acquisition at 83°C for 6 sec.

The melting peaks of all samples were routinely determined by melting curve analysis in order to ascertain that only the expected products had been generated. Additionally, the length of all PCR products was monitored by agarose gel electrophoresis analysis (3% agarose, ethidium bromide stained). Cloned PCR products of each of the respective transcripts were used to generate external standard curves. Routinely, dilutions of standards covering five orders of magnitude (5 × 10^-16 ^to 5 × 10^-12 ^g DNA/reaction) were freshly diluted from stocks of 10 ng DNA/μl and co-amplified during each run. Copy numbers were calculated relative to the amount of total RNA previously subjected to cDNA synthesis. To normalize for variations between individual LightCycler runs one or two arbitrarily selected samples were co-amplified as calibrators.

### Statistical analysis

Line-specific means of component fertility traits and of transcript abundance levels were compared with t-testing. When p < 0.05, differences between lines were considered significant. Correlation analysis comparing microarray and qPCR data was performed with the Pearson Product Moment correlation test. Relationships between pairs of variables were considered significant when p < 0.05. All tests were calculated with the SigmaStat software (Jandel Scientific, San Rafael, CA, USA)

## Authors' contributions

JV, GN and NR conceived the experimental design. ML was responsible for animal breeding, line selection and sampling. DK conducted RNA preparation, labelling and microarray hybridization and together with GN conducted the statistical evaluation of raw data. H–JT enabled and supervised microarray analysis and critically read the manuscript. JV conducted the qPCR re-analysis, interpreted the results and drafted the manuscript. All authors approved the final manuscript.

## Supplementary Material

Additional file 1 Differentially expressed transcripts identified by microarray analysis

## References

[B1] Monget P, Fabre S, Mulsant P, Lecerf F, Elsen JM, Mazerbourg S, Pisselet C, Monniaux D (2002). Regulation of ovarian folliculogenesis by IGF and BMP system in domestic animals. Domestic Animal Endocrinology.

[B2] Clutter AC, Kirby YLK, Nielsen MK (1994). Uterine Capacity and Ovulation Rate in Mice Selected 21 Generations on Alternative Criteria to Increase Litter Size. Journal of Animal Science.

[B3] Gion JM, Clutter AC, Nielsen MK (1990). Alternative methods of selection for litter size in mice: II. Response to thirteen generations of selection. Journal of Animal Science.

[B4] Holl JW, Robison OW (2003). Results from nine generations of selection for increased litter size in swine. Journal of Animal Science.

[B5] Kirby YK, Nielsen MK (1993). Alternative methods of selection for litter size in mice: III. Response to 21 generations of selection. Journal of Animal Science.

[B6] Ruiz-Flores A, Johnson RK (2001). Direct and correlated responses to two-stage selection for ovulation rate and number of fully formed pigs at birth in swine. Journal of Animal Science.

[B7] Spitschak M, Langhammer M, Schneider F, Renne U, Vanselow J (2007). Two high-fertility mouse lines show differences in component fertility traits after long-term selection. Reproduction, Fertility and Development.

[B8] Jefferson WN, Couse JF, Padilla-Banks E, Korach KS, Newbold RR (2002). Neonatal exposure to genistein induces estrogen receptor (ER)alpha expression and multioocyte follicles in the maturing mouse ovary: Evidence for ER beta-mediated and nonestrogenic actions. Biol Reprod.

[B9] Lucci CM, Amorim CA, Rodrigues APR, Figueiredo JR, Bao SN, Silva JRV, Goncalves PBD (1999). Study of preantral follicle population in situ and after mechanical isolation from caprine ovaries at different reproductive stages. Animal Reproduction Science.

[B10] Tilly JL, Tilly KI, Perez GI (1997). The genes of cell death and cellular susceptibility to apoptosis in the ovary: a hypothesis. Cell Death & Differentiation.

[B11] Valdez KE, Cuneo SP, Turzillo AM (2005). Regulation of apoptosis in the atresia of dominant bovine follicles of the first follicular wave following ovulation. Reproduction.

[B12] Yacobi K, Tsafriri A, Gross A (2007). Luteinizing hormone-induced caspase activation in rat preovulatory follicles is coupled to mitochondrial steroidogenesis. Endocrinology.

[B13] de Kretser DM, Robertson DM (1989). The isolation and physiology of inhibin and related proteins. Biol Reprod.

[B14] Sicinski P, Donaher JL, Geng Y, Parker SB, Gardner H, Park MY, Robker RL, Richards JS, McGinnis LK, Biggers JD, Eppig JJ, Bronson RT, Elledge SJ, Weinberg RA (1996). Cyclin D2 is an FSH-responsive gene involved in gonadal cell proliferation and oncogenesis. Nature.

[B15] Pradeep PK, Li X, Peegel H, Menon KM (2002). Dihydrotestosterone inhibits granulosa cell proliferation by decreasing the cyclin D2 mRNA expression and cell cycle arrest at G1 phase. Endocrinology.

[B16] Luciano AM, Goudet G, Perazzoli F, Lahuec C, Gerard N (2006). Glutathione content and glutathione peroxidase expression in in vivo and in vitro matured equine oocytes. Mol Reprod Dev.

[B17] Al-Gubory KH, Ceballos-Picot I, Nicole A, Bolifraud P, Germain G, Michaud M, Mayeur C, Blachier F (2005). Changes in activities of superoxide dismutase, nitric oxide synthase, glutathione-dependent enzymes and the incidence of apoptosis in sheep corpus luteum during the estrous cycle. Biochim Biophys Acta.

[B18] Leyens G, Verhaeghe B, Landtmeters M, Marchandise J, Knoops B, Donnay I (2004). Peroxiredoxin 6 is upregulated in bovine oocytes and cumulus cells during in vitro maturation: role of intercellular communication. Biol Reprod.

[B19] Spagnuolo MS, Cigliano L, Balestrieri M, Porta A, Abrescia P (2001). Synthesis of ascorbate and urate in the ovary of water buffalo. Free Radical Research.

[B20] Tsuji M, Ito Y, Terada N, Mori H (1989). Ovarian aromatase activity in scorbutic mutant rats unable to synthesize ascorbic acid. Acta Endocrinol (Copenh).

[B21] Takahashi T, Fukuda K, Pan J, Kodama H, Sano M, Makino S, Kato T, Manabe T, Ogawa S (1999). Characterization of insulin-like growth factor-1-induced activation of the JAK/STAT pathway in rat cardiomyocytes. Circulation Research.

[B22] Clyne CD, Speed CJ, Zhou J, Simpson ER (2002). Liver receptor homologue-1 (LRH-1) regulates expression of aromatase in preadipocytes. Journal of Biological Chemistry.

[B23] Sirianni R, Seely JB, Attia G, Stocco DM, Carr BR, Pezzi V, Rainey WE (2002). Liver receptor homologue-1 is expressed in human steroidogenic tissues and activates transcription of genes encoding steroidogenic enzymes. Journal of Endocrinology.

[B24] Falender AE, Lanz R, Malenfant D, Belanger L, Richards JS (2003). Differential expression of steroidogenic factor-1 and FTF/LRH-1 in the rodent ovary. Endocrinology.

[B25] Labelle-Dumais C, Pare JF, Belanger L, Farookhi R, Dufort D (2007). Impaired Progesterone Production in Nr5a2+/- Mice Leads to a Reduction in Female Reproductive Function. Biol Reprod.

[B26] Chen YJ, Hsiao PW, Lee MT, Mason JI, Ke FC, Hwang JJ (2007). Interplay of PI3K and cAMP/PKA signaling, and rapamycin-hypersensitivity in TGFbeta1 enhancement of FSH-stimulated steroidogenesis in rat ovarian granulosa cells. Journal of Endocrinology.

[B27] Guastavino JM, Boufares S, Crusio WE (2005). Ovarian abnormalities in the staggerer mutant mouse. Scientific World Journal.

[B28] Jo M, Curry TE (2006). Luteinizing hormone-induced RUNX1 regulates the expression of genes in granulosa cells of rat periovulatory follicles. Molecular Endocrinology.

[B29] Wood JR, Nelson VL, Ho C, Jansen E, Wang CY, Urbanek M, McAllister JM, Mosselman S, Strauss JF (2003). The molecular phenotype of polycystic ovary syndrome (PCOS) theca cells and new candidate PCOS genes defined by microarray analysis. Journal of Biological Chemistry.

[B30] Luo CW, Kawamura K, Klein C, Hsueh AJ (2004). Paracrine regulation of ovarian granulosa cell differentiation by stanniocalcin (STC) 1: mediation through specific STC1 receptors. Molecular Endocrinology.

[B31] Chang AC, Cha J, Koentgen F, Reddel RR (2005). The murine stanniocalcin 1 gene is not essential for growth and development. Mol Cell Biol.

[B32] Kendziorski C, Irizarry RA, Chen KS, Haag JD, Gould MN (2005). On the utility of pooling biological samples in microarray experiments. Proc Natl Acad Sci U S A.

[B33] Schüler L (1985). Selection for Fertility in Mice - the Selection Plateau and How to Overcome It. Theoretical and Applied Genetics.

[B34] Dietl G, Langhammer M, Renne U (2004). Model simulations for genetic random drift in the outbred strain Fzt : DU. Archives of Animal Breeding.

[B35] Schüler L, Bünger L (1982). The Reproductive Lifetime Performance of Laboratory Mouse Lines Selected for Fertility. Archives of Animal Breeding.

[B36] Storey JD, Tibshirani R (2003). Statistical significance for genomewide studies. Proc Natl Acad Sci U S A.

[B37] Storey JD (2002). A direct approach to false discovery rates. Journal of the Royal Statistical Society.

