# Supplementary material for: Expression profiling of a high-fertility mouse line by microarray analysis and qPCR
Source: BMC Genomics. 2008 Jun 27;9:307. doi: 10.1186/1471-2164-9-307 (PMC2443385; doi:10.1186/1471-2164-9-307)
Supplement: Additional file 1 — Differentially expressed transcripts identified by microarray analysis [file 1471-2164-9-307-s1.doc]

Additional file 1: Differentially expressed transcripts identified by microarray analysis

| **%** | **Probe Set ID** | **Gene Symbol** | **Gene Title** | **FC** |
| --- | --- | --- | --- | --- |
| 1.4 | *Apoptosis* |  |  |  |
|  | 1427819_at | *Bcl2* | B-cell CLL/lymphoma 2 | -2,9 (-) |
|  | 1415995_at | *Casp6* | caspase 6 | 1,6 (***) |
| 1.4 | Oocyte/follicle development | |  |  |
|  | 1426859_at | *Inhbb* | inhibin beta-B | -1,7 (***) |
|  | 1417542_at | *Rps6ka2* | ribosomal protein S6 kinase, polypeptide 2 | 1,8 |
| 3.4 | Cell cycle |  |  |  |
|  | 1418003_at | *1190002H23Rik* | RIKEN cDNA 1190002H23 gene | 2,8 |
|  | 1448229_s_at | *Ccnd2* | cyclin D2 | -2,2 (***) |
|  | 1438852_x_at | *Mcm6* | minichromosome maintenance deficient 6 | -1,8 |
|  | 1438390_s_at | *Pttg1* | pituitary tumor-transforming 1 | -1,9 |
|  | 1448005_at | *Sash1* | SAM and SH3 domain containing 1 | -1,7 |
| 3.4 | Oxidative stress_oxygen transport | | |  |
|  | 1449106_at | *Gpx3* | glutathione peroxidase 3 | 1,9 |
|  | 1451297_at | *Gulo* | gulonolactone (L-) oxidase | -1,8 |
|  | 1452757_s_at | *Hba-a1* | hemoglobin alpha, adult chain 1 | 1,6 |
|  | 1417184_s_at | *Hbb-b1* | hemoglobin, beta adult major chain, minor chain | 1,8 |
|  | 1416292_at | *Prdx3* | peroxiredoxin 3 | 1,8 |
| 4.1 | Ion transport |  |  |  |
|  | 1451152_a_at | *Atp1b1* | ATPase, Na+/K+ transporting, beta 1 polypeptide | 1,8 |
|  | 1417343_at | *Fxyd6* | FXYD domain-containing ion transport regulator 6 | -2,6 |
|  | 1448696_at | *Heph* | hephaestin | -1,7 |
|  | 1426633_s_at | *Kctd14* | potassium channel tetramerisation domain containing 14 | -2,9 |
|  | 1418445_at | *Slc16a2* | solute carrier family 16, member 2 | 1,5 |
|  | 1448482_at | *Slc39a8* | solute carrier family 39, member 8 | -1,6 |
| 4.8 | Immune response | |  |  |
|  | 1451593_at | *H2-K1* | Histocompatibility 2, K1, K region | 3,0 |
|  | 1418536_at | *H2-Q7* | Histocompatibility 2, Q region locus 7 | 4,3 (***) |
|  | 1452348_s_at | *Ifi203* | interferon activated gene 203 | 7,9 |
|  | 1452349_x_at | *Ifi205* | interferon activated gene 205 | 9,0 (***) |
|  | 1460258_at | *Lect1* | leukocyte cell derived chemotaxin 1 | 4,9 |
|  | 1420603_s_at | *Raet1a* | retinoic acid early transcript 1 | 6,5 (***) |
|  | 1434438_at | *Samhd1* | SAM domain and HD domain, 1 | 2,4 |
| 6.1 | Cell adhesion/interaction & cytoskeleton | | |  |
|  | 1418815_at | *Cdh2* | cadherin 2 | -2,7 (***) |
|  | 1453473_a_at | *Dynlt1* | dynein light chain Tctex-type 1 | 1,7 |
|  | 1426756_at | *Galnt2* | UDP-N-acetyl-alpha-D-galactosamine:polypeptide N-acetylgalactosaminyltransferase 2 | -2,0 |
|  | 1449408_at | *Jam2* | junction adhesion molecule 2 | -2,3 |
|  | 1417079_s_at | *Lgals2* | lectin, galactose-binding, soluble 2 | 4,4 |
|  | 1425510_at | *Mark1* | MAP/microtubule affinity-regulating kinase 1 | -1,5 |
|  | 1452670_at | *Myl9* | myosin, light polypeptide 9 | -1,5 |
|  | 1419339_at | *Neu3* | neuraminidase 3 | -4,2 |
|  | 1416342_at | *Tnc* | tenascin C | 1,7 |
| 6.8 | Protein/amino acid metabolism/transport & proteolysis | | |  |
|  | 1450624_at | *Bhmt* | betaine-homocysteine methyltransferase | 3,4 |
|  | 1425623_a_at | *Cbs* | cystathionine beta-synthase | -2,4 |
|  | 1417233_at | *Chchd4* | coiled-coil-helix-coiled-coil-helix domain containing 4 | -2,2 |
|  | 1418365_at | *Ctsh* | cathepsin H | -1,5 |
|  | 1448433_a_at | *Pcolce* | procollagen C-endopeptidase enhancer protein | 1,6 |
|  | 1421556_at | *Serpina3a* | serine peptidase inhibitor, clade A, member 3A | -2,2 |
|  | 1421564_at | *Serpina3c* | serine peptidase inhibitor, clade A, member 3C | 4,1 |
|  | 1418436_at | *Stx7* | syntaxin 7 | 1,8 |
|  | 1427477_at | *Tmprss13* | transmembrane protease, serine 13 | -2,3 |
|  | 1448848_at | *Tor1b* | torsin family 1, member B | -1,6 |
| 9.5 | Intracellular signal transduction | |  |  |
|  | 1456307_s_at | *Adcy7* | adenylate cyclase 7 | -1,6 |
|  | 1429692_s_at | *Gch1* | GTP cyclohydrolase 1 | -1,8 |
|  | 1454696_at | *Gnb1* | guanine nucleotide binding protein, beta 1 | -2,3 |
|  | 1419469_at | *Gnb4* | guanine nucleotide binding protein, beta 4 | 1,6 |
|  | 1420533_at | *Gucy1a3* | guanylate cyclase 1, soluble, alpha 3 | -1,9 |
|  | 1420872_at | *Gucy1b3* | guanylate cyclase 1, soluble, beta 3 | -1,8 |
|  | 1459894_at | *Iqgap2* | IQ motif containing GTPase activating protein 2 | -2,2 |
|  | 1420411_a_at | *Pi4k2b* | phosphatidylinositol 4-kinase type 2 beta | -3,5 (-) |
|  | 1420349_at | *Ptgfr* | prostaglandin F receptor | 3,3 (***) |
|  | 1425284_a_at | *Rab27a* | RAB27A, member RAS oncogene family | 2,4 |
|  | 1423619_at | *Rasd1* | RAS, dexamethasone-induced 1 | -1,9 |
|  | 1420941_at | *Rgs5* | regulator of G-protein signaling 5 | -2,0 (-) |
|  | 1422140_at | *Sp100-rs* | similar to component of Sp100-rs | -2,5 |
|  | 1416770_at | *Stk25* | serine/threonine kinase 25 (yeast) | -2,8 |
| 10.2 | Steroid/lipid metabolism | |  |  |
|  | 1418601_at | *Aldh1a7* | aldehyde dehydrogenase family 1, subfamily A7 | -2,4 |
|  | 1448804_at | *Cyp11a1* | cytochrome P450, family 11, subfamily a, polypeptide 1 | 1,9 (***) |
|  | 1416612_at | *Cyp1b1* | cytochrome P450, family 1, subfamily b, polypeptide 1 | 1,5 |
|  | 1419367_at | *Decr1* | 2,4-dienoyl CoA reductase 1, mitochondrial | 1,7 |
|  | 1429076_a_at | *Gdpd2* | glycerophosphodiester phosphodiesterase domain containing 2 | -3,4 |
|  | 1431833_a_at | *Hmgcs2* | 3-hydroxy-3-methylglutaryl-Coenzyme A synthase 2 | 3,3 |
|  | 1448865_at | *Hsd17b7* | hydroxysteroid (17-beta) dehydrogenase 7 | 3,2 |
|  | 1426516_a_at | *Lpin1* | lipin 1 | -2,4 |
|  | 1448513_a_at | *Npc2* | Niemann Pick type C2 | 1,5 |
|  | 1420410_at | *Nr5a2* | nuclear receptor subfamily 5, group A, member 2 | -1,6 (**) |
|  | 1424390_at | *Nupl1* | nucleoporin like 1 | -1,9 |
|  | 1425083_at | *Otor* | otoraplin | -2,7 |
|  | 1451457_at | *Sc5d* | sterol-C5-desaturase | 2,1 |
|  | 1417697_at | *Soat1* | sterol O-acyltransferase 1 | 3,8 |
|  | 1426258_at | *Sorl1* | sortilin-related receptor, LDLR class A repeats-containing | -2,4 |
| 15.0 | Regulation of transcription/chromatin/translation | | |  |
|  | 1419979_s_at | *Creb3* | cAMP responsive element binding protein 3 | 2,4 |
|  | 1420427_a_at | *Dhx32* | DEAH (Asp-Glu-Ala-His) box polypeptide 32 | 7,9 |
|  | 1417182_at | *Dnaja2* | DnaJ (Hsp40) homolog, subfamily A, member 2 | -1,5 |
|  | 1460562_at | *Eftud1* | elongation factor Tu GTP binding domain containing 1 | 1,6 |
|  | 1437159_at | *Eif2c5* | eukaryotic translation initiation factor 2C, 5 | 5,9 |
|  | 1420491_at | *Eif2s1* | eukaryotic translation initiation factor 2, subunit 1 alpha | 8,3 |
|  | 1416101_a_at | *Hist1h1c* | histone 1, H1c | 1,6 |
|  | 1425868_at | *Hist2h2bb* | Histone 2, H2bb | -3,1 |
|  | 1419363_a_at | *Mrpl35* | mitochondrial ribosomal protein L35 | 1,7 |
|  | 1425349_a_at | *Myef2* | myelin basic protein expression factor 2, repressor | -2,4 |
|  | 1421431_at | *Ptrf* | polymerase I and transcript release factor | -1,9 |
|  | 1426604_at | *Rnasel* | ribonuclease L (2', 5'-oligoisoadenylate synthetase-dependent) | 3,7 |
|  | 1424034_at | *Rora* | RAR-related orphan receptor alpha | 1,9 |
|  | 1422128_at | *Rpl14* | ribosomal protein L14 | -2,0 |
|  | 1422864_at | *Runx1* | runt related transcription factor 1 | -1,6 (-) |
|  | 1418467_at | *Smarcd3* | SWI/SNF related, matrix associated, actin dependent regulator of chromatin, subfamily d, member 3 | 1,7 |
|  | 1448753_at | *Srp9* | signal recognition particle 9 | 1,5 |
|  | 1420915_at | *Stat1* | signal transducer and activator of transcription 1 | 1,8 (***) |
|  | 1436382_at | *Zbtb12* | zinc finger and BTB domain containing 12 | 1,6 |
|  | 1433705_at | *Zfp213* | zinc finger protein 213 | -2,1 |
|  | 1451146_at | *Zfp386* | zinc finger protein 386 (Kruppel-like) | 2,3 |
|  | 1453198_at | *Zfp422-rs1* | zinc finger protein 422, related sequence 1 | -1,9 |
| 35.0 | Others & unknown | |  |  |
|  | 1455539_at | --- | --- | -2,4 |
|  | 1455965_at | --- | --- | 5,6 |
|  | 1431008_at | *0610037M15Rik* | RIKEN cDNA 0610037M15 gene | 3,3 |
|  | 1428922_at | *1200009O22Rik* | RIKEN cDNA 1200009O22 gene | 1,5 |
|  | 1424072_at | *2010107G23Rik* | RIKEN cDNA 2010107G23 gene | 2,0 |
|  | 1452705_at | *2210010A19Rik* | RIKEN cDNA 2210010A19 gene | -1,9 |
|  | 1438082_at | *2310028N02Rik* | RIKEN cDNA 2310028N02 gene | -1,7 |
|  | 1423672_at | *2510042P03Rik* | RIKEN cDNA 2510042P03 gene | -1,6 |
|  | 1423266_at | *2810405K02Rik* | RIKEN cDNA 2810405K02 gene | 1,7 |
|  | 1454632_at | *6330442E10Rik* | RIKEN cDNA 6330442E10 gene | 1,9 |
|  | 1439272_at | *A830039H10Rik* | RIKEN cDNA A830039H10 gene | -2,7 |
|  | 1439483_at | *AI506816* | expressed sequence AI506816 | -9,1 |
|  | 1442028_at | *AI593864* | expressed sequence AI593864 | -2,6 |
|  | 1433935_at | *AU020206* | expressed sequence AU020206 | 1,6 |
|  | 1423122_at | *Avpi1* | arginine vasopressin-induced 1 | 2,5 |
|  | 1419490_at | *AW049604* | expressed sequence AW049604 | -2,0 |
|  | 1454984_at | *AW061234* | expressed sequence AW061234 | 2,1 |
|  | 1438278_a_at | *BC003993* | cDNA sequence BC003993 | -7,3 |
|  | 1425046_at | *BC018465* | cDNA sequence BC018465 | -2,2 |
|  | 1457147_at | *BC026657* | cDNA sequence BC026657 | -2,8 |
|  | 1439422_a_at | *C1qdc2* | C1q domain containing 2 | 4,4 |
|  | 1450080_at | *Cxx1c* | CAAX box 1 homolog C (human) | -2,5 |
|  | 1449273_at | *Cyfip2* | cytoplasmic FMR1 interacting protein 2 | -1,6 |
|  | 1417821_at | *D17H6S56E-5* | DNA segment, Chr 17, human D6S56E 5 | -1,5 |
|  | 1418298_s_at | *Dpysl4* | dihydropyrimidinase-like 4 | 1,9 |
|  | 1434292_at | *E130013N09Rik* | RIKEN cDNA E130013N09 gene | 7,1 |
|  | 1420965_a_at | *Enc1* | ectodermal-neural cortex 1 | -2,0 |
|  | 1417203_at | *Ethe1* | ethylmalonic encephalopathy 1 | 2,3 |
|  | 1438700_at | *Fnbp4* | formin binding protein 4 | -1,7 |
|  | 1452907_at | *Galc* | galactosylceramidase | 6,6 |
|  | 1420342_at | *Gdap10* | ganglioside-induced differentiation-associated-protein 10 | 2,0 |
|  | 1449859_at | *Golt1b* | golgi transport 1 homolog B (S. cerevisiae) | 7,2 |
|  | 1421041_s_at | *Gsta1/Gsta2* | glutathione S-transferase, alpha 1 (Ya) | -1,8 |
|  | 1424609_a_at | *LOC432823* | similar to hypothetical protein MGC37588 | -2,0 |
|  | 1446165_at | *LOC621312* | similar to fibrillarin | 2,4 |
|  | 1455749_x_at | *Ndufa7* | NADH dehydrogenase (ubiquinone) 1 alpha subcomplex, 7 | 1,9 |
|  | 1422126_a_at | *Nudt13* | nudix (nucleoside diphosphate linked moiety X)-type motif 13 | 3,8 |
|  | 1417928_at | *Pdlim4* | PDZ and LIM domain 4 | 2,0 |
|  | 1418760_at | *Rdh11* | retinol dehydrogenase 11 | 4,1 |
|  | 1451204_at | *Scara5* | scavenger receptor class A, member 5 (putative) | -1,8 |
|  | 1419588_at | *Spag1* | sperm associated antigen 1 | 2,3 |
|  | 1416114_at | *Sparcl1* | SPARC-like 1 (mast9, hevin) | -1,7 |
|  | 1450448_at | *Stc1* | stanniocalcin 1 | -1,9 |
|  | 1438910_a_at | *Stom* | stomatin | -1,8 |
|  | 1422882_at | *Sypl* | synaptophysin-like protein | -2,8 |
|  | 1424454_at | *Tmem87a* | transmembrane protein 87A | 8,6 |
|  | 1426641_at | *Trib2* | tribbles homolog 2 (Drosophila) | -1,7 |
|  | 1416689_at | *Tuft1* | tuftelin 1 | -2,5 |
|  | 1454881_s_at | *Upk3b* | uroplakin 3B | 2,5 |
|  | 1418486_at | *Vnn1* | vanin 1 | 8,0 |
|  | 1451006_at | *Xdh* | xanthine dehydrogenase | 2,0 |

%, percentage of transcripts mapping to specific categories of biological processes relative to the total number of differentially expressed transcripts; FC, fold change; (*), (**), and (***), transcripts with significantly different line-specific expression levels (FL1 vs. control line) in qPCR experiments (p<0.05, p< 0.01, and p< 0.001, respectively, t-test); transcripts significantly (p<0.05, Chi square test) mapping to GO terms of biological processes are underlined.
